# Mechanism and Therapeutic Opportunities of Histone Modifications in Chronic Liver Disease

**DOI:** 10.3389/fphar.2021.784591

**Published:** 2021-11-23

**Authors:** Qiuyu Cai, Can Gan, Chengwei Tang, Hao Wu, Jinhang Gao

**Affiliations:** ^1^ Laboratory of Gastroenterology and Hepatology, West China Hospital, Sichuan University, Chengdu, China; ^2^ Department of Gastroenterology, West China Hospital, Sichuan University, Chengdu, China

**Keywords:** histone acetylation, histone methylation, histone phosphorylation, alcoholic liver disease, metabolic associated fatty liver disease, viral hepatitis, liver fibrosis, liver cirrhosis

## Abstract

Chronic liver disease (CLD) represents a global health problem, accounting for the heavy burden of disability and increased health care utilization. Epigenome alterations play an important role in the occurrence and progression of CLD. Histone modifications, which include acetylation, methylation, and phosphorylation, represent an essential part of epigenetic modifications that affect the transcriptional activity of genes. Different from genetic mutations, histone modifications are plastic and reversible. They can be modulated pharmacologically without changing the DNA sequence. Thus, there might be chances to establish interventional solutions by targeting histone modifications to reverse CLD. Here we summarized the roles of histone modifications in the context of alcoholic liver disease (ALD), metabolic associated fatty liver disease (MAFLD), viral hepatitis, autoimmune liver disease, drug-induced liver injury (DILI), and liver fibrosis or cirrhosis. The potential targets of histone modifications for translation into therapeutics were also investigated. In prospect, high efficacy and low toxicity drugs that are selectively targeting histone modifications are required to completely reverse CLD and prevent the development of liver cirrhosis and malignancy.

## 1 Introduction

Chronic liver disease (CLD) and its ultimate outcome liver cirrhosis represent a heavy global health burden, accounting for two million deaths each year (Mokdad et al., 2014; Tapper and Parikh, 2018). The incidence and prevalence rates of CLD are rising worldwide (Liangpunsakul et al., 2016; Wong et al., 2019; Younossi, 2019; Moon et al., 2020), resulting in a high rate of disability and increased health care utilization (Moon et al., 2020). The etiologies of CLD comprise chronic Hepatitis B virus (HBV) and Hepatitis C virus (HCV) infection, metabolic syndrome, excessive alcohol consumption, exposure to the chemical compound, autoimmune response, *etc*., (Zeng et al., 2021). These etiologies contribute to chronic liver damages, which can progress to liver fibrosis, liver cirrhosis, and even hepatocellular carcinoma (HCC) (Hardy and Mann, 2016), further reducing the life quality of patients. Nowadays, HCV infection can be cured with antiviral treatments, and HBV infection can be prevented by vaccines and suppressed by oral antiviral regimens (Younossi et al., 2020). However, therapeutic options for CLD remain insufficient in many cases (Zeidler et al., 2017). It is essential to elucidate the mechanism in the occurrence and progression of CLD and develop new effective therapeutics.

Epigenetic modifications include DNA methylation, histone modifications, non-coding regulatory RNA-mediated processes, and chromatin remodeling, which regulate gene expression without changing DNA sequences (Chen et al., 2020). In recent years, emerging studies on epigenetics have provided novel insights into the pathogenesis and treatment of CLD (Wilson et al., 2017). This review mainly focuses on the roles and therapeutic opportunities of histone modifications in CLDs, particularly in alcoholic liver disease (ALD), metabolic-associated fatty liver disease (MAFLD), viral hepatitis, and liver fibrosis/cirrhosis. The role of histone modification in HCC has been summarized elsewhere (Khan et al., 2017; Han et al., 2018; Rajan et al., 2020; Wolinska and Skrzypczak, 2021), and is not the focus of the current review.

## 2 Histones and Histone Modifications

### 2.1 Histones

**FIGURE 1:**
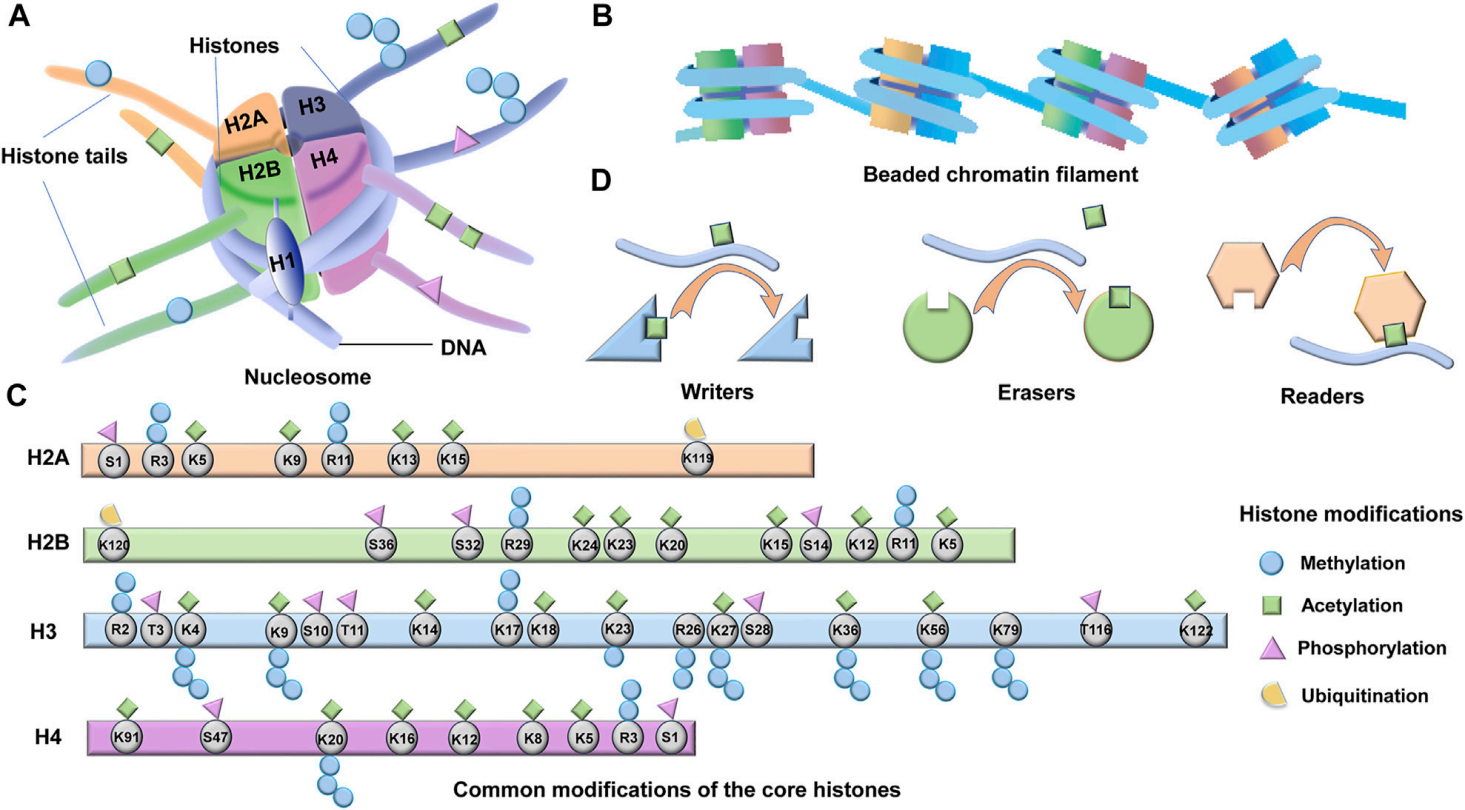
Structure and modifications of histones. **(A)** Four core histone proteins (H2A, H2B, H3, and H4) and one linker histone H1 which binds to the site where DNA enters and exits the nucleosome core particle, form a complete nucleosome. Post-translational modifications (PTMs) occur on histone terminal tails, which are accessible on the surface of the nucleosome. **(B)** The nucleosomes are linked by a sequence of DNA (∼60 bp) and are assembled into beaded chromatin filaments. **(C)** The potential acetylation, methylation, phosphorylation, and ubiquitination sites of the four core histones are shown. **(D)** A large array of proteins are involved in regulating and interpreting these PTMs, including readers, writers, and erasers.

Histones are highly conserved essential proteins and are the main protein components of chromatin in all eukaryotic cells (Strahl and Allis, 2000; Shechter et al., 2007). Two major types of histone proteins, core histones (H2A, H2B, H3, and H4) and the linker histone (H1), play crucial roles in genome packaging. At the initial stage of DNA packaging, two turns of DNA (∼150 bp) are wrapped around an octamer composed of dimers of the four core histone proteins (H2A, H2B, H3, and H4) to form the nucleosome core particle, the recurring unit structure of chromatin (Figure 1A) (Kamakaka and Biggins, 2005; Kawashima et al., 2015). One molecule of linker histone H1 binds to the site where DNA enters and exits the nucleosome core particle, forming a complete nucleosome (Figure 1A) (Fyodorov et al., 2018). The nucleosomes are linked by a sequence of DNA (∼60 bp) and are assembled into beaded chromatin filaments (Figure 1B) (Lawrence et al., 2016; Fyodorov et al., 2018). At the advanced level of DNA packaging, the filaments are folded into fibers and eventually packaged into the nucleus (Figure 1B). In this process, histone H1 contributes to chromatin compaction and the formation of higher-order chromatin structures (Ponte et al., 2017).

### 2.2 Histone Modifications

The four core histones (H2A, H2B, H3, and H4) contain a conserved C- terminal histone fold structure and a flexible N- terminal tail protruding from the nucleosome (Figure 1C) (Kamakaka and Biggins, 2005). The linker histones (H1) instead consist of a short, unique N- terminal tail, a central stably folded domain, and a long, intrinsically disordered C- terminal tail (Allan et al., 1980). Histones impede transcription by condensing DNA and physical obstruction (Lorch et al., 1987; Kouzarides, 2007). However, histones have more elaborate functions depending on the post-translational modifications (PTMs) of their terminal tails, which are accessible on the surface of the nucleosome (Figures 1A,C) (Li et al., 2007; Allis and Jenuwein, 2016). PTMs of terminal histones, including acetylation, methylation, phosphorylation, ubiquitination, sumoylation and ADP-ribosylation, can directly influence protein stability, localization, activity, and the interactions between histone-DNA and histone-histone (Apweiler et al., 2004; Vu et al., 2018). The cumulative effect of multiple histone modifications ultimately determines the transcriptional activity of a gene. The histone tails and the common PTMs sites are shown in Figure 1C. In addition to the abovementioned effects, histone modifications can also affect the recruitment of chromatin modifiers as well as transcription factors (Ausio et al., 1989; Clements et al., 2003). Moreover, histone modifications can recruit effector proteins and indirectly activate downstream signaling (Wysocka et al., 2006). Despite the diversity of modification types, histone modifications and their corresponding regulating enzymes represent significant specificity during residue modification and epigenome (Bates, 2020). There are hundreds of enzymes regulating the PTMs of histones, and these enzymes are categorized as writers, readers, or erasers according to their effects on chromatin function (Figure 1D) (Tarakhovsky, 2010; Bates, 2020). As the name implies, “writers” are enzymes that add modifications, “readers” are bromodomain and chromodomain proteins that recognize acetylated or methylated residues, whereas “erasers” are enzymes that remove post-translational modifications (Figure 1D). The writer, reader, and eraser proteins regulate gene expression *via* histone modifications and eventually involve in the development of CLD. In this review, we focus on the functions and regulations of the three best-studied types of PTMs in CLD, which are acetylation, methylation, and phosphorylation of terminal histones.

#### 2.2.1 Histone Acetylation

Histone acetylation is modulated via the balance of the activity of histone acetyltransferases (HATs) and histone deacetylases (HDACs) (Guo et al., 2018). HATs catalyze the reaction where an acetyl group from the acetyl-CoA transfer to the ε-amino group on histone lysine residues (Daskalaki et al., 2018). Nevertheless, HDACs catalyze deacetylation by hydrolyzing acetyl groups from the lysine residues (Schneider et al., 2013). Acetylation of histones can neutralize the positive charges of histones and decrease electrostatic interactions between the histones and DNA, since the DNA is in a negative charge. Thus, histone acetylation relaxes histone-DNA interactions and turns the chromatin to a transcriptionally active state (Hardy and Mann, 2016). On the contrary, deacetylation of histones leads to the formation of heterochromatin and represses the transcriptional activity of DNA (Guo et al., 2018). In terms of the structure and functional properties, the “writer” protein HATs can be divided into five categories: p300/CBP, Gcn5-related N-acetyltransferases (GNAT), SRC, MYST, and TAFII250 (Gajer et al., 2015). Besides, the “erasers” protein HDACs can be classified into four subclasses: Class I-IV HDACs. Class I HDACs, including HDAC1, HDAC2, HDAC3, and HDAC8, are associated with the RPD3 deacetylase; class II HDACs contains HDAC4-10 and are homologous to the histone deacetylase 1 (HDA1); class III HDACs, including sirtuin 1 (SIRT1) to SIRT7, represent with homology to silent information regulator 2 (Sir2); and Class IV (HDAC11) (Seo et al., 2014; Guo et al., 2018). Moreover, histone acetylation is recognized by bromodomain readers. Bromodomain-containing protein 4 (BRD4), as an acetylation reader and a member of the bromodomain and extraterminal (BET) family (Peverelli et al., 2017), reads the hyperacetylated regions of chromatin (Stathis et al., 2016).

#### 2.2.2 Histone Methylation

The methylation of histones is regulated by the activity of histone methyltransferases (HMTs) and histone demethylases (HDMTs) (Kim et al., 2021). HMTs catalyze the addition of methyl groups from S-adenosylmethionine (SAM) onto lysine and arginine residues of histones. Unlike HATs, HMTs methylate not only histones, but also non-histone proteins. On the contrary, HDMTs catalyze the elimination of methyl groups from histones (Greer and Shi, 2012). Lysine residues have three methylation states: mono- (me), di- (me2) and trimethylation (me3), while arginine residues have different patterns of methylation: monomethylated (me) or symmetrically dimethylated (me2s) or asymmetrically dimethylated (me2a) (Gong and Miller, 2019). Different from other modifications of histones, histone methylation occurs mainly on the lysine or arginine residues of H3 and H4 (Moghe et al., 2011), and represents different effects on transcription depending on the location and state of the specific residue being modified. For instance, methylation on H3K9, H3K27, and H4K20 has a silencing effect, while methylation on H3K4, H3K36, and H3K79 has an activating effect (Santos-Rosa et al., 2002; Black et al., 2012; Collins et al., 2019). HMTs are divided into three categories: SET-domain containing enzymes, Dot1-like proteins acting on lysine (KMTs), and arginine N-methyltransferase enzymes (PRMTs) acting on arginine (Black et al., 2012; Blanc and Richard, 2017). Whereas, lysine demethylases (KDMs) are classified into two families: amine oxidases and iron-dependent dioxygenases with a jumonji C (JmjC)-domain (Shi et al., 2004; Tsukada et al., 2006; Whetstine et al., 2006). The characterization of arginine demethylase is not well understood, although a subset of JmjC KDMs, deaminase enzymes peptidyl arginine deaminase 4 (PAD4), and Jumonji domain-containing protein 6 (JMJD6) have been reported to demethylate arginines (Chang et al., 2007; Walport et al., 2016). Methylation of histones does not change the electronic charge of the histones, nor does it change the DNA-histones interactions. Therefore, histone methylation might mainly function by influencing the binding of specific “readers” to the methylated site (Martin and Zhang, 2005). These “readers” are methyl binding motifs containing chromodomain proteins, including the chromobox (CBX) family and chromodomain helicase DNA binding protein (CHD1) (Hyun et al., 2017; Bates, 2020).

#### 2.2.3 Histone Phosphorylation

Histone phosphorylation status is mediated by the activity of kinases and phosphatases. Kinases add phosphate groups from ATP mainly to the hydroxyl group on serine, threonine, and tyrosine residues of histones, while phosphatases remove the phosphates from these residues (Bannister and Kouzarides, 2011; Rossetto et al., 2012). The addition of remarkably negative charges and changed histone structures due to histone phosphorylation might induce chromatin relaxation (Roque et al., 2008; Lopez et al., 2015). Besides, histone phosphorylation interacts with other histone modifications, and the cross-talk regulates the chromatin status and interactions between them (Zippo et al., 2009). It is known that histone phosphorylation plays a role in DNA damage and repair, compaction of chromatin related to mitosis and meiosis, and the modulation of transcription (Alaskhar Alhamwe et al., 2018). Phosphorylation is recognized by “readers”, which are proteins containing phospho-binding modules, such as 14-3-3 and BRCT domains (Yun et al., 2011). The “readers” are also characterized as downstream effectors.

### 2.3 Experimental Methods for Histone Modification Detection

Chromatin immunoprecipitation (ChIP) test is the most traditional strategy in studying DNA-protein interactions (Kuo and Allis, 1999). In this procedure, chromatin proteins are temporarily cross-linked to DNA, and the harvested chromatin is sheared into several short DNA-protein parts (Chenarani et al., 2021). Selected sections containing proteins of interest are subsequently immunoprecipitated by incorporating antibodies specific to these proteins. The protein-bound DNA fragments are then purified and sequenced by numerous analytical methods (Decaprio and Kohl, 2020). The result is a rundown of short DNA fragments attached by specific proteins (Chenarani et al., 2021). However, the technique requires optimization of reaction conditions, and its application is limited by the sparse reads or low cell throughput. Therefore, ChIP is often implied in combination with other techniques.

Re-ChIP methods, which employ sequential immunoprecipitation reactions, were developed to identify multiple proteins bound to a single DNA sequence (Truax and Greer, 2012). However, the yield of Re-ChIP is very low with great fluctuation. In addition, ChIP, coupled with microarrays (ChIP–chip), enables the DNA sections obtained from ChIP to be identified by hybridization to microarrays, therefore providing genome-wide profiling of DNA-histone interactions (Park, 2009). Similarly, ChIP, combined with next-generation sequencing technologies (ChIP-Seq), also allows the genome-wide identification of histone binding sites, and has more excellent coverage, higher resolution, and less noise (Park, 2009). As for the classical ChIP experiment, many studies have proposed improvement schemes of experimental steps to increase its practicability, resulting in improved methods including scChIP-seq, scChIL-seq, scChIC-seq, iscChIC-seq, *etc* (Rotem et al., 2015; Harada et al., 2019; Ku et al., 2019; Ku et al., 2021). Reviews of these methods are also available for readers who have interests (Ludwig and Bintu, 2019; Ma et al., 2019; Harada et al., 2021).

## 3 Histone Modifications in CLDs

### 3.1 Alcoholic Liver Disease (ALD)

ALD is a kind of CLD caused by chronic and excessive alcohol use. ALD can first be presented as simple steatosis (fat accumulation in hepatocyte), and then progress to alcoholic hepatitis (steatosis concurrent with inflammation), liver fibrosis, cirrhosis, and even HCC (Cao et al., 2021). Alcohol is first oxidized primarily in the cytoplasm of hepatocytes by alcohol dehydrogenase (ADH) (Osna et al., 2017). The ADH-catalyzed ethanol oxidation consumes nicotinamide adenine dinucleotide (NAD+), and generates reduced NAD+ (NADH) as well as acetaldehyde. Acetaldehyde and its adducts are highly reactive and toxic. However, acetaldehyde is rapidly oxidized to acetate under the catalysis of aldehyde dehydrogenase 2 (ALDH2) inside mitochondria, accompanied with NAD+ consumption and NADH production (Osna et al., 2017). The pathogenesis of ALD is quite complex and results from the interaction of multiple mechanisms (Lin et al., 2018; Sehrawat et al., 2020). The metabolism of ethanol in the liver produces abundant reactive oxygen species (ROS) and alters the balance between lipogenesis and fatty acid metabolism, leading to hepatic steatosis (Xu et al., 2017). Besides, alteration of the NADH/NAD^+^ ratio, mitochondrial function impairment caused by the metabolite acetaldehyde, gut microbial-derived lipopolysaccharides (LPS) translocation caused by increased intestinal permeability, and excessive inflammatory responses also contribute to the development of ALD (Seitz et al., 2018; Bajaj, 2019; Cao et al., 2021). Interestingly, ALDH2, which is crucial in acetaldehyde detoxification, has been reported to play a beneficial role in attenuating chronic alcohol-induced hepatic carbonyl formation, hepatic oxidative stress, apoptosis, and regulation of autophagy, thereby ameliorating hepatic steatosis and inflammation induced by chronic alcohol intake (Guo et al., 2009; Guo et al., 2015). Therefore, the genetic polymorphism of ALDH2 may also play a crucial role in the pathogenesis of ALD. Apart from these pathological mechanisms, histone modifications in intrahepatic cells, mainly including acetylation, methylation, and phosphorylation of histones, were also demonstrated to be involved in the development of ALD (Kriss et al., 2018).

#### 3.1.1 Histone Acetylation in ALD

**FIGURE 2:**
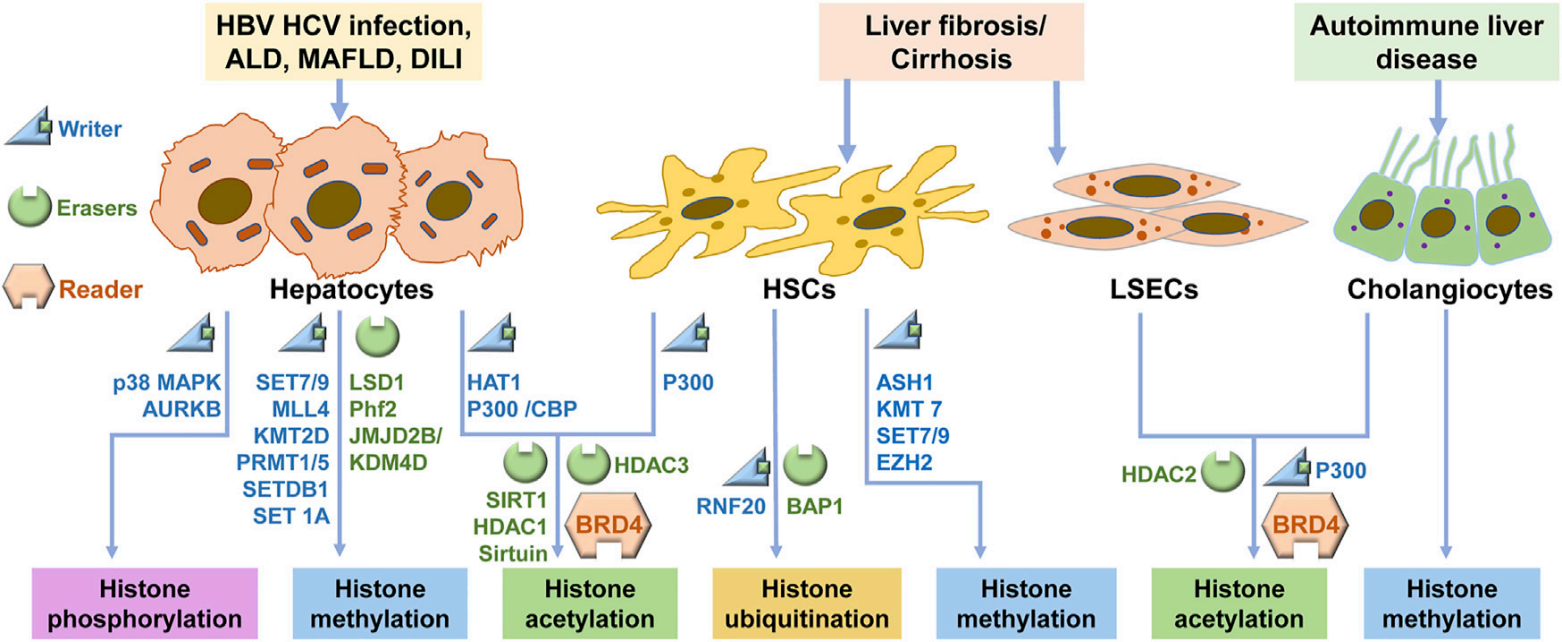
Signaling regulations of histone modification in CLDs. In CLD, hepatocytes, HSCs, LSECs and cholangiocytes undergo histone modifications via the regulation of the writers and erasers, and these modifications are recognized by readers. ALD: alcoholic liver disease; MAFLD: metabolic-associated fatty liver disease; DILI: Drug-induced liver injury; HSC: hepatic stellate cell; LSEC: liver sinusoidal endothelial cell.

**FIGURE 3:**
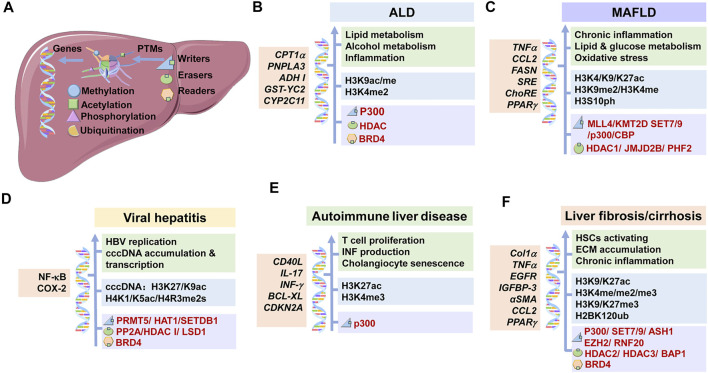
Underlying mechanisms of histone modifications in CLDs. The main histone modifications, the targeted genes, the corresponding effects and the therapeutic targets in CLDs were shown. **(A)** Modifications of histones are mediated via writers and erasers, and are recognized by readers. These modifications work through changing the expression of corresponding genes. **(B)** In ALD, p300, HDAC, and BRD4 mediate the histone modifications at specific genes, which regulate lipid and alcohol metabolisms and inflammation reaction. **(C)** In MAFLD, p300/CBP, HDAC1, SET7/9 *et al.* mediate the histone modifications at specific genes, which promote chronic inflammation, lipid and glucose metabolism and oxidative stress. **(D)** In viral hepatitis, PRMT5, HDAC1, BRD4 *et al.* mediate the histone modifications at specific genes, which regulate HBV replication, cccDNA accumulation and transcription. **(E)** In autoimmune liver disease, p300 and other proteins mediate the histone modifications at specific genes, which promote T cell proliferation, INF production and cholangiocyte senescence. **(F)** In liver fibrosis/cirrhosis, p300, SET7/9, HDAC 3 *et al.* mediate the histone modifications at specific genes, which promote HSC activation, ECM accumulation and chronic inflammation. ALD: alcoholic liver disease; MAFLD: metabolic-associated fatty liver disease; HSC: hepatic stellate cell; IFN: interferon; ECM: extracellular matrix.

It was reported that ethanol selectively increased acetylation of Lys9 in histone H3 (H3K9) in a dose-dependent and time-dependent manner in rat hepatocytes *in vitro* (Park et al., 2003). In addition, chronic ethanol treatment in rats selectively increases the class I alcohol dehydrogenase (ADH I) gene expression via enhancing H3K9 acetylation in promoter and coding regions of *ADH I* (Park et al., 2012). Besides, alcohol-induced acetylation was site-specific, mainly on H3K9/K14 and H3K18/K23 (Kriss et al., 2018). These reports imply that there might be a disease-relevant epigenetic signature in ALD, and alcohol intake may induce the expression of *ADH I*, thereby accelerating the oxidation of ingested ethanol to acetaldehyde. Furthermore, increased H3K9 acetylation by HDAC3 inhibitor Trichostatin A (TSA) permits the expression of the β-oxidation gene carnitine palmitoyltransferase 1α (CPT1α) and attenuates binge alcohol-induced hepatic steatosis (Kirpich et al., 2013). Moreover, acetylation of H3K9 at the promoter of patatin-like phospholipase domain-containing 3 (PNPLA3) increases expression of the lipid homeostasis-related gene *PNPLA3* (Restrepo et al., 2017). These results suggest that alcohol intake promotes the acetylation of H3K9 at lipogenesis genes (*e.g*., *PNPLA3*) and inhibits the acetylation of H3K9 at lipolysis genes (*e.g*., CPT1α), which increases lipid anabolism and reduces lipid catabolism, respectively, thus breaking the homeostasis of fats in ALD. Acetylation of histones is mainly accomplished via activating HATs and inhibiting HDACs (Table 1; Figure 2) (Park et al., 2005; Huber et al., 2007). For example, p300 (a subfamily of HATs) and H3K9 acetylation levels were elevated simultaneously at peak blood alcohol levels in rats with chronic ethanol intake (Bardag-Gorce et al., 2007). Besides, many studies have demonstrated that the expression of HDAC is inhibited and is associated with increased hepatic histone acetylation after alcohol exposure (Yeung et al., 2004; You et al., 2008; Kirpich et al., 2012). In addition to the deacetylation function, SIRT1 promotes the conversion of the metabolite of ethanol acetic acid into acetyl-CoA, which acetylates histones under the catalysis of HATs (Sahar et al., 2014; Lin et al., 2018). Therefore, SIRT1 may be involved in the balance of gene silencing and activation in ALD *via* acetylation of histones. However, few studies focus on the role of SIRT in ALD, which should be especially studied in the future. Additionally, inhibition of histone acetylation reader BRD4 either by epigenome editing or selective inhibitor iBET-151 could suppress the chemokine expression in liver sinusoidal endothelial cells (LSECs) and reduce neutrophil infiltration in murine models of alcoholic hepatitis (Figure 2) (Liu et al., 2021). These results imply that histone acetylation are indeed involved in the inflammatory response of alcoholic hepatitis, and that the role of histone acetylation depends to some extent on the involvement of readers. In summary, histone acetylation involves ALD via up- or down-regulating the transcription of *ADH I*, *CPT*1α, and *PNPLA3* under the regulation of writers, erasers, and readers, and sequently promoting alcohol metabolism, accumulation of fats, and hepatic inflammation (Figure 3B).

**TABLE 1:** Summary of histone modifications and the corresponding mechanisms involved in CLD.

Diseases	PTM site	Corresponding effect	Writer/Eraser/inhibitor	References
ALD	H3K9ac	Activation of *CPT1α*, *ADH I*, *PNPLA3*	p300/HDAC/TSA	Park et al. (2012), Kirpich et al. (2013), Restrepo et al. (2017)
	H3K9/14/18/23ac and H3K27me3	—	—	Bardag-Gorce et al. (2009), Kriss et al. (2018)
	H3K4me2	Activation of *ADH I* and *GST-Yc2*	—	Pal-Bhadra et al. (2007)
	H3K9me	Repression of *Lsdh* and *CYP2C11*	—	Pal-Bhadra et al. (2007)
	H3S10/28ph	Activating H3K14 acetylation and inhibting acetylation of H3K9	p38 MAPK	Lee and Shukla (2007)
MAFLD	H3K9/K14/K18ac	Activation of *TNFα*, *CCL2*, Pol2 and NF-κB recruitment	HDAC1	Aagaard-Tillery et al. (2008), Mikula et al. (2014)
	H3K4/K9ac and H4K8/K16/K20ac and H3S10ph	Activation of *FASN*, *SRE*, *ChoRE*, *PPARγ*, *SREBP*-*1c*, *ACLY*	HAT (p300/CBP)	Du et al. (2017), Cai et al. (2018), Chung et al. (2019), Rohrbach et al. (2019)
	H3K27ac	Activation of *Scd1*, *Cyp4a14*, *PPAR*, *C/EBP*, *NF4*, and *SREBP*	---	Siersbæk et al. (2017), Becares et al. (2019)
	H3K9me2/3 demethylation	Activation of *LXRE* and *ERO1-α*, increasing hepatic LXRα-dependent lipogenic genes	JMJD2B	Li et al. (2012), Kim et al. (2020)
	H3K9me2 demethylation	Activation of *ChREBP*, *PPARγ*, *Nrf2*, or *HIF1α*, and protecting the liver from pathogenic lipids and ROS accumulation	KDM (Phf2)	Bricambert et al. (2018)
	H3K4me	Activating steatotic target genes of *PPARγ2* and TNFα-induced inflammatory genes	MLL4/KMT2D/SET7/9	Li et al. (2008), Kim et al. (2016)
Viral hepatitis	H2A.Zac and H3K9ac deacetylation	Changes in hepatocyte chromatin structure	Sirtuin deacetylase	Jenke et al. (2014)
	H4 deacetylation and decreased H2AXph	Changing hepatocarcinogenesis-associated genes expression and impeding DNA damage repair	PP2A	Duong et al. (2010)
	H3K27/H4K5/H4K12ac on cccDNA	Promoting HBV replication and cccDNA accumulation	HAT1/HDAC I	Pollicino et al. (2006), Luo et al. (2013), Koumbi et al. (2016), Yang et al. (2019)
	H3K9/K27 deacetylation in cccDNA	Required for cccDNA transcription	HDAC	Liu et al. (2013)
	H4 demethylation	Changing expression of genes important for hepatocarcinogenesis	PRMT1	Duong et al. (2010)
	H3K27me3 demethylation	—	—	Hlady et al. (2021)
	H3K9me and demethylation of H3K4me3 in cccDNA	Recruitment of HP1 and consequently repressed viral gene expression	SETDB1, SET 1A/LSD1	Rivière et al. (2015), Alarcon et al. (2016)
	H4R3me2s on cccDNA	Repressing cccDNA transcription	PRMT5	Zhang et al. (2017)
	Decreased H3S10ph	Downregulation of NF-κB and COX-2 transcription	AURKB	Madejón et al. (2015)
	Decreased H3ph in cccDNA	Reducing replication, transcription, and antigen secretion of HBV	—	Luo et al. (2013)
Autoimmune liver disease	H4 acetylation	Activation of *LIGHT*, *CD40L*, *IL-17* and *INF-γ*, increasing T cell proliferation, interferon production	—	Hu et al. (2011)
	H4 deacetylation	Activation of *HDAC7A*, *APO2*, and *TRAIL*, reducing activities of NF-κB and AP-1	—	Hu et al. (2011)
	H3K27ac	Activation of anti-apoptotic *BCL-xL* during cholangiocyte senescence	p300	O’hara et al. (2019)
	H3K4me3	Activation of *CDKN2A* during cholangiocyte senescence	—	O’hara et al. (2017)
DILI	H3K9ac	Activation of stress-related genes	—	Sinha et al. (2016)
	H3K9me3	Repression of stress and metabolic-associated genes	—	Sinha et al. (2016)
	H3S10ph	An critical role in liver regeneration after injury	—	Sinha et al. (2016)
Liver fibrosis/Cirrhosis	H3K9ac	Activation of *TNFα* and *EGFR*, increases MYC and cyclin D1, activating HSCs	—	Yang et al. (2014), Sarr et al. (2016), Zheng et al. (2020)
	H3K27ac	Activation of *AP-1*, *Col1α1/2*, *TEAD*, *IGFBP-3* and NF-κB, and activating HSCs, activation of TNFα-induced CCL2 transcription in LSECs	p300/BRD4	Liu et al. (2020), Yaqoob et al. (2020) Gao et al. (2021)
	H4 deacetylation/acetylation	Repression of *PPARγ* gene during cirrhosis/inhibiting HSCs proliferation and collagen deposition	HDAC3/HDACi (VPA)	(Mannaerts et al., 2010; Rodríguez-Aguilera et al., 2018)
	H3K4me/me3	Activation of *αSMA*, *TIMP*, *Col1* and *TGF-β1*/increasing HIF-1 nuclear transport, autophagosome formation, and activation of HSC	ASH1	Perugorria et al. (2012), Hong et al. (2018)
	Increased H3K4me1/2/3, reduced H3K9me2/3	TGF-β overexpression	KMT 7 (SET7/9)	Sheen-Chen et al. (2014)
	H3K27me3	Repression of *PPARγ*, *CFTR*, enhancing autophagy	Methyltransferas EZH2	Zeybel et al. (2012), Yang et al. (2018)
	H2BK120ub	Repression of *IL-6*, *TNF-α*, *VEGFA*, *α-SMA* and *Col1α1*	RNF20	Chen et al. (2021)
	H2A deubiquitination	Apoptosis of fibroblasts and liver tissue	BAP1	He et al. (2019)

Abbreviations: Alcohol dehydrogenase I (ADHI); Alpha-smooth muscle actin (αSMA); Angiopoietin-like 4 (Angtpl4); ATP-citrate lyase (ACLY); Aurora B kinase (AURKB); Carbohydrate-responsive elements (ChoRE); Carbohydrate-responsive element-binding protein (ChREBP); Carnitine palmitoyltransferase-1alpha (CPT1alpha); Chemokine C-C ligand 2 (CCL2); Cyclin-dependent kinase inhibitor 2A (CDKN2A); Cystic fibrosis transmembrane conductance regulator (CFTR); Cytochrome P450, family 4, subfamily a, polypeptide 14 (Cyp4a14); Fatty acid synthase (FASN); Fibroblast growth factor 21 (Fgf21); Glutathione-S-transferase subunit Yc2 (GST Yc2); HDAC3 inhibitor Trichostatin A (TSA); Heterochromatin protein 1 factor (HP1); Histone lysine demethylase-1 (LSD1); Homocysteine (Hcy); Hypoxia-inducible factor-1 (HIF-1); Insulin receptor substrate 2 (Irs2); Insulin-like growth factor binding protein-3 (IGFBP-3); Lactate dehydrogenase (LDH). Nuclear factor, interleukin 3 regulated (Nfil3); Nuclear factor-κB (NF-κB); Patatin-like phospholipase domain-containing 3 (PNPLA3); Peroxisome proliferator activated receptor (PPAR); Plant Homeodomain Finger 2 (Phf2); Polymerase 2 RNA (POL2); Protein phosphatase 2A (PP2A); Pyruvate dehydrogenase complex (PDHC); Sterol regulatory elements (SRE); Sterol-regulatory element binding protein- (SREBP); Synectin (GIPC); Tissue inhibitor of metalloproteinase-1 (TIMP1); Transforming growth factor (TGF-β) Transcription factor NF-E2-related factor 2 (Nrf2); Tumor necrosis factor alpha (TNFα); ring finger protein 20 (RNF20); BRCA1-Associated Protein 1 (BAP1);

#### 3.1.2 Histone Methylation in ALD

In addition to histone acetylation, histone methylation also plays a critical role in the process of ALD (Benevolenskaya, 2007). Alcohol administration reduced the dimethylation of H3K9 and increased the dimethylation of H3K4 in primary rat hepatocytes (Pal-Bhadra et al., 2007). Besides, increased trimethylation of H3K27 was found in a rat model with chronic ethanol administration (Bardag-Gorce et al., 2009). According to these results, the level and types of histone methylation are indeed altered in ALD, suggesting that histone methylation may be involved in the occurrence and development of ALD. Further studies have shown that accumulated H3K9 methylation at the promoter was related to ethanol-downregulated genes like L-serine dehydratase (LSDH) and Cytochrome P450 2C11 (CYP2C11) (Pal-Bhadra et al., 2007). Nevertheless, increased methylation of H3K4 and reduced H3K9 methylation in the gene regulatory region were associated with ethanol-upregulated genes ADH I and Glutathione S-transferase Yc2 (GST-Yc2) (Pal-Bhadra et al., 2007). These results further confirm that alcohol promotes locus-specific chromatin modification, and the alcohol-induced changes in gene expression might be regulated through histone modification (Figure 3B). In addition, S-adenosyl-L-methionine (SAM), an important molecule in histone methylation, is mainly derived from the liver and is metabolized to S-adenosylhomocysteine (SAH) by methyltransferases (Mato et al., 2002). SAM is the major methyl donor of histone methylation, while SAH is an effective histone methylation inhibitor (Fontecave et al., 2004; Lu and Mato, 2008). Accumulated evidence reveals that ethanol causes hepatic SAM deficiency via various mechanisms (Ara et al., 2008; Bardag-Gorce et al., 2010; Moghe et al., 2011). In addition, alcohol administration followed by SAM treatment can attenuate the alcohol-induced liver injury (Ara et al., 2008; Bardag-Gorce et al., 2010; Moghe et al., 2011). These studies imply that alcohol might affect the methylation process of histones *via* SAM metabolism, and SAM supplementation may reverse histone demethylation induced by alcohol. However, it remains unclear how histone methylation directly takes part in the pathogenesis of ALD, and further studies are needed.

#### 3.1.3 Histone Phosphorylation in ALD

Phosphorylation of histones is also involved in the development of ALD along with other modifications of histones. Ethanol and its metabolite increased phosphorylation of histone H3 at serine 10 (H3S10) and serine 28 (H3S28) in primary rat hepatocytes via activation of p38 mitogen-activated protein kinase (MAPK) (Table 1; Figure 2) (Lee and Shukla, 2007; Aroor et al., 2010). Moreover, phosphorylation of H3S10 was able to activate the acetylation of H3K14 and inhibit the acetylation of H3K9 *in vitro* (Lo et al., 2000; Edmondson et al., 2002). Therefore, it is plausible that ethanol affects the modification of histones in both independent and orchestrated ways (Shukla et al., 2015). Little is known about the specific role of histone phosphorylation in ALD, and more researches are still urgently needed. In general, chronic alcohol intake may lead to histone acetylation, methylation, and phosphorylation, which affect the expression of relevant genes, leading to the progression of ALD. However, further research is needed to fill in the gaps in the role of histone modifications in pathogenesis (Figure 3B).

### 3.2 Metabolic Associated Fatty Liver Disease (MAFLD)

Nonalcoholic fatty liver disease (NAFLD) is becoming the leading cause of end-stage CLD and liver failure (Kazankov et al., 2019). NAFLD ranges from simple steatosis to its progressive and inflammatory form, nonalcoholic steatohepatitis (NASH) (Lee et al., 2017; Abdelmalek, 2021). NAFLD is assumed to be associated with obesity status and metabolic dysfunctions, such as dyslipidemia, insulin resistance (IR), diabetes mellitus, cardiovascular disease, etc., (Ipsen et al., 2018). Therefore, experts recently reached a consensus to rename NAFLD to metabolic dysfunction associated fatty liver disease (MAFLD), which is a more appropriate overarching term (Eslam et al., 2020). Lipid acquisition in the liver depends on the uptake of circulating lipids and *de novo* lipogenesis, while lipid disposal in the liver depends on fatty acid oxidation and lipids export. The imbalance between lipid acquisition and lipid disposal finally results in hepatic steatosis (Ipsen et al., 2018). Mechanisms underlying the pathological steatosis of the liver are not fully elucidated. Currently, abnormal hepatic lipid metabolism, oxidative stress response, mitochondrial damage, and inflammatory cytokines releasement are proved to be involved in the pathogenesis of MAFLD (Manne et al., 2018). Moreover, emerging evidence suggests that MAFLD is an epigenetics-driven disease in which various epigenetic mechanisms mediate gene-environment interactions (Sun et al., 2015; Eslam et al., 2018). Here, we mainly focus on the role of histone modifications in MAFLD.

#### 3.2.1 Histone Acetylation in MAFLD

Histone acetylation is the most commonly studied histone modification, and acetylation of H3 and H4 is the most known alterations in MAFLD (Du et al., 2017; Morral et al., 2021). In chronic high-fat maternal diet-induced fetal Japanese macaques model, HDAC1 was downregulated in the liver and associated with significant hyperacetylation of H3K14 and increased acetylation at H3K9 and H3K18 in liver tissue (Table 1; Figure 2) (Aagaard-Tillery et al., 2008). These results demonstrated that a high-fat maternal diet led to acetylation of histones in fetal, and the epigenetic change acquired in fetal could serve as origins of adult disease. Besides, obese mice had an increase in H3K9/K18ac at coding regions of tumor necrosis factor α (*T*NFα) and chemokine C-C motif ligand two genes (*CCL2*), which are key inflammatory mediators in MAFLD (Table 1) (Mikula et al., 2014). Moreover, H3K9/K18ac level was increased at *TNF*α and *CCL2* in Hepa1-6 cells after treatment with lipopolysaccharide (LPS), resulting in polymerase two RNA (*POL2*) and nuclear factor-κB (NF-κ*B*) recruitment (Table 1) (Mikula et al., 2014). This gives us a clue that the acetylation of H3K9/K18 induced by obesity can activate the transcription of pro-inflammatory-related genes, triggering or aggravating the inflammatory responses in MAFLD. With insulin stimulation, H3, H4 hyperacetylation (H3K4, H3K9, and H4K20) was found in the promoter of fatty acid synthase (*FASN*), sterol regulatory elements (*SRE*), and carbohydrate-responsive elements (*ChoRE*) (Table 1), which function in the *de novo* of lipogenesis, cholic acid synthesis, and cholesterol absorption, and lipogenic and glycolytic, respectively (Du et al., 2017; Cai et al., 2018; Rohrbach et al., 2019). Similarly, H4K8ac and H4K16ac were also accompanied by increased HAT activity in HepG2 cells with lipid accumulation (Table 1; Figure 2) (Chung et al., 2019). The above studies suggest IR may enhance acetylation of histone H3 and H4 at lipogenesis-related genes, which might be responsible for the fat accumulation in hepatic steatosis. Additionally, H3K27ac is an established marker for active regulatory elements in promoters and enhancers (Zhang et al., 2013). It was reported that H3K27ac is up-regulated at the enhancers of related genes in high-fat diet mice (Table 1), and the H3K27ac level can be reversed by weight loss (Siersbæk et al., 2017; Becares et al., 2019). In conclusion, histone acetylation, serving as a tool for gene transcription regulation, takes part in the development of MAFLD, and these epigenetic changes in histone acetylation are highly dynamic and reversible (Figure 3C).

#### 3.2.2 Histone Methylation in MAFLD

Aberrant histone methylation profile is also involved in the process of MAFLD. Liver X receptors (LXRs) are nuclear receptors for oxysterols. After binding of the ligand, LXR would translocate to the nucleus and activate the transcription of genes containing LXR response elements (*LXREs*) (Slominski et al., 2021). In MAFLD, the repressive histone marks (H3K9me2 and H3K9me3) near LXRE in the promoter region of LXRα-target genes were removed by histone demethylase Jumonji domain-containing protein 2B (JMJD2B) (Table 1; Figure 2) (Kim et al., 2020). These alterations lead to increased expression of hepatic LXRα-dependent lipogenic genes, which contribute to the development of hepatic steatosis (Kim et al., 2020). Besides, transgenerational high-fat diet (HFD) feeding reduced H3K9me2 accumulation at promoters of *LX*Rα and endoplasmic reticulum oxidoreductin-alpha (ERO1-α) in the offspring mice, which account for the up-regulation of lipogenesis and endoplasmic reticulum (ER) stress in the development of obesity and hepatic steatosis (Li et al., 2012). Besides, in another study, H3K9me2 demethylation mediated by histone demethylase plant homeodomain finger 2 (Phf2) at the promoter of carbohydrate responsive element-binding protein (ChREBP)-regulated genes increases transcription of the corresponding genes (Table 1; Figure 2) (Bricambert et al., 2018). ChREBP is a transcription factor that governs glycolytic and lipogenic genes. Activated ChREBP activates glycolysis and fatty acid synthesis and inhibits lipolysis (Linden et al., 2018). Therefore, H3K9me2 demethylation at the promoter of ChREBP facilitates lipogenesis in the liver. However, H3K9me2 demethylation at promoter of NF-E2-related factor 2 (Nrf2) reroutes glucose fluxes toward the pentose phosphate pathway and promotes glutathione biosynthesis, protecting the liver from pathogenic ROS accumulation and fibrogenesis in the MAFLD progression (Table 1; Figure 2) (Bricambert et al., 2018). These results imply that H3K9me2 may be decreased at specific gene promoters, thereby promoting adipogenesis and protecting liver from the pathogenesis progression, and these epigenetic changes accumulate over generations (Figure 3C).

In addition to H3K9, H3K4 methylation also plays a pivotal role in the development of MAFLD. Peroxisome proliferator-activated receptor-gamma 2 (PPARγ2) is a transcription factor for hepatic steatosis induced by overnutrition. In a murine MAFLD model, overnutrition contributes to hepatic steatosis by facilitating H3K4 methylation *via* H3K4 methyltransferase MLL4/KMT2D at steatotic target genes of PPARγ2 and activating their transcription (Table 1; Figure 2) (Kim et al., 2016). Moreover, methylation of H3K4 on the promoters of TNFα-induced inflammatory genes by methyltransferase SET7/9 affects the expression of TNFα-induced inflammatory cytokines (Table 1; Figure 2) (Li et al., 2008). These results provide us with a clue that H3K4 methylation activates the transcription of specific genes, which contributes to hepatic steatosis and induces inflammation response, leading to progression to steatohepatitis (Figure 3C).

#### 3.2.3 Histone Phosphorylation in MAFLD

Although less well studied, histone phosphorylation still plays a unique role in MAFLD. As previously mentioned, MAFLD is closely associated with diabetes mellitus, which is characterized by elevated blood glucose levels and abnormal insulin levels or function (Manne et al., 2018). Higher H3S10 phosphorylation and H3 and H4 acetylation within the promoter and exon 2 regions of *ChORE* and *FASN* were observed in high glucose treated HepG2 and L02 cells (Table 1) (Cai et al., 2018). H3S10 phosphorylation might interact with other histone modifications to influence the transcriptional activity of genes (Figure 3C). However, the specific role of histone phosphorylation in MAFLD remains unclear and needs more investigations in the future. In conclusion, acetylation, methylation and phosphorylation of histones are involved in the process of MAFLD, which affects the expression of specific genes, resulting in the progression of MAFLD. However, more profound research is still needed to create a complete map of histone modifications in MAFLD and to understand their roles in the pathogenesis and progression of MAFLD (Figure 3C).

### 3.3 Viral Hepatitis (Hepatitis B and Hepatitis C)

Viral hepatitis is the leading cause of cirrhosis-related mortality in Asia (Moon et al., 2020). There are five types of hepatitis (A, B, C, D, and E), caused by five kinds of hepatitis virus, respectively. Chronic viral hepatitis occurs after the infection of HBV, HCV, hepatitis D virus (HDV), and occasionally hepatitis E virus (HEV). In this review, we mainly focus on cases with chronic infection of HBV and HCV. HBV is a covalently closed circular double-stranded DNA virus, and HCV is a positive-sense single-stranded RNA virus (Lanini et al., 2019). Both HBV and HCV are non-cytopathic viruses. Therefore, their pathogenesis is mainly regulated by metabolic changes and viral protein-induced host immunity (Irshad et al., 2013). Besides, viruses can induce changes in genetic sequence and the epigenetic status of host liver cells. HBV can integrate into the host genome or persist as a minichromosome of covalently closed circular DNA (cccDNA) with histone and non-histone proteins (Koumbi and Karayiannis, 2015). Persistent cccDNA existence and immune tolerance to HBV antigens in the liver are responsible for the chronic infection of HBV (Shih et al., 2018). The mechanisms of HCV persistence remain incompletely characterized, while viral assaulting on the host innate immune system and the defective adaptive immunity are included (Li et al., 2015). The chronic infection of viruses can lead to the host-specific immune-mediated liver damage and progression to cirrhosis and HCC (Seeger and Mason, 2015; Singal et al., 2020). Here, we discuss the changes in histone modifications after the infection of HBV or HCV and their roles in the disease progression.

#### 3.3.1 Histone Acetylation in Viral Hepatitis

Histone modification occurs not only on the chromosome of host liver cells, but also on histones of HBV minichromosome. In HBV-infected cells, most of the abovementioned PTMs changes are induced by Hepatitis B virus X protein (HBx), an important protein for HBV replication (Luo et al., 2013). Hypoacetylation of H2A.Z and H3K9 was found in the chromosome of HBV infected hepatocytes owing to the elevated sirtuin deacetylase activity, resulting in chromatin structure changes (Table 1; Figure 2) (Jenke et al., 2014). Similarly, inhibition of histone H4 acetylation was also found in HCV-infected cells *in vitro*, which changed the expression of crucial genes for hepatocarcinogenesis (Table 1) (Duong et al., 2010). These studies prove that the acetylation status of histones in hepatocytes is changed after virus infection, leading to changes in chromatin structure and expression of specific genes. Besides, HAT1-catalyzed acetylation of H3K27/H4K5/H4K12 in cccDNA was found to promot HBV replication and cccDNA accumulation in a human liver-chimeric mouse model (Yang et al., 2019). On the contrary, hypoacetylated cccDNA-associated H3/H4 histones were accompanied by HDAC I recruitment in liver tissue and were correlated with low HBV replication (Pollicino et al., 2006; Luo et al., 2013; Koumbi et al., 2016). In addition, HDAC I inhibitors induced a significant increase in cccDNA-bound acetylated H4 and HBV replication (Table 1; Figure 2) (Pollicino et al., 2006). Moreover, a study of duck hepatitis B virus (DHBV) *in vitro* demonstrated that deacetylation of H3K9 and H3K27 in cccDNA minichromosome was associated with the suppression of cccDNA transcription (Table 1) (Liu et al., 2013). These observed results imply that acetylation of cccDNA-associated histones facilitates HBV replication as well as cccDNA accumulation and transcription. Taken together, histone acetylation alters the epigenome of both the virus and the host liver cells, which in turn affects viral and host gene replication and transcription (Figure 3D). However, in-depth studies are needed to explain the effect and the underlying mechanisms of histone acetylation in the pathogenesis of viral hepatitis.

#### 3.3.2 Histone Methylation in Viral Hepatitis

Histone methylation is a common epigenetic change both in viruses and the host cells in viral hepatitis. Inhibition of histone H4 methylation was found in genes essential for hepatocarcinogenesis with changed expression in HCV proteins-induced cell lines, which might be responsible for the hepatocarcinogenesis in chronic hepatitis C (Table 1) (Duong et al., 2010). In addition, a decrease of H3K27me3, a silencing mark of transcription, was found in HCV-infected cells (Hlady et al., 2021). These studies indicate that alterations in histone methylation status in hepatocytes after viruses infection regulate the expression of genes which may exacerbate disease progression. For viruses, *in vitro* studies found that methylation of H3K9 and demethylation of H3K4me3 on viral promoters accompanied with the recruitment of heterochromatin protein 1 factor (HP1) is correlated with condensed chromatin and repressed viral gene expression (Rivière et al., 2015; Alarcon et al., 2016). The increase of H3K9me3 was mainly mediated by SETDB1, a type of histone methyltransferase, and the demethylation of H3K4me3 was mainly mediated by LSD1, a type of histone lysine demethylase (Table 1; Figure 2) (Rivière et al., 2015). Moreover, symmetric dimethylation of arginine 3 in H4 (H4R3me2s) on cccDNA, which was mediated by arginine methyltransferase 5 (PRMT5), was reported to have a repressive effect on cccDNA transcription (Table 1; Figure 2) (Zhang et al., 2017). These *in vitro* studies imply that the influence of cccDNA histone methylation on viral transcription resembles the impact of chromosome histone methylation on gene translation in eukaryotic cells. At present, cccDNA-associated histone methylation is found to affect the transcription of the viral gene. However, its effect on viral replication is still unknown. *In vivo*, H3K4me3 was related to viral transcription and patient HBeAg status in specimens from HBV patients (Flecken et al., 2019). However, cccDNA-associated H3K9me3 (usually a silencing mark) was not linked to decreased viral transcription (Flecken et al., 2019). These results show that the epigenetic landscape of chronic HBV infection is more complex *in vivo*, and *in vitro* models cannot fully simulate the situation *in vivo* (Figure 3D).

#### 3.3.3 Histone Phosphorylation in Viral Hepatitis

Phosphorylation of histones is much less studied but still plays a role in viral hepatitis. In a previous study, phosphorylation of H3S10 was inhibited in human primary hepatocytes infected with HCV or transfected with HCV core protein (Madejón et al., 2015). The authors assumed that inhibition of H3S10 was due to the direct interaction between HCV core and Aurora B kinase (AURKB), which also induced a decrease of AURKB activity and downregulation of NF-κB and *COX-2* transcription (Table 1; Figure 2) (Madejón et al., 2015). These changes may regulate the inflammatory response at the initial phase of viral infection, ensuring HCV infectivity. Besides, inhibition of histone H2AX phosphorylation and H4 methylation/acetylation was reported in HCV-infected cells, which might change the expression of hepatocarcinogenesis-associated genes and impede DNA damage repair (Table 1) (Duong et al., 2010). In an *in vitro* study of HBV infection, a decrease in phosphorylated, methylated, and acetylated cccDNA-bound histone H3 was found in HBx mutant HBV-infected cells, resulting in reduced replication, transcription, and antigen secretion of HBV (Table 1) (Luo et al., 2013). From those abovementioned studies, we cannot conclude the specific role of histone phosphorylation in the development of viral hepatitis. Histone phosphorylation often cooperates with other modifications to participate in the development of disease. There are few studies on the effect of hitone phosphorylation in viral hepatitis, and more studies are needed to explore the underlying mechanisms. In summary, acetylation, methylation and phosphorylation of histones participate in the process of viral replication, and affect the expression of specific genes of viruses as well as host cells. However, further studies are still needed to reveal the underlying mechanisms of histone modifications in viral hepatitis.

### 3.4 Autoimmune Liver Disease

Autoimmune liver diseases mainly comprise primary biliary cholangitis (PBC), autoimmune hepatitis (AIH), and primary sclerosing cholangitis (PSC) (Floreani et al., 2019). Till now, there lacks curative treatment for all three disorders (Carbone and Neuberger, 2014). Therefore, it is necessary to understand the underlying pathophysiological mechanisms and develop new therapies. PBC is characterized as the destruction of the primary bile ducts by antimitochondrial antibodies and autoreactive T cells (Hu et al., 2011). In PBC, the multifunctional signaling molecule β-Arrestins is crucial to T cell survival (Hu et al., 2011). β-Arrestin-1 promoted H4 acetylation in the promoter regions of *LIGHT*, *CD40L*, interleukin 17 (*IL-17*), and interferon-γ (IFNγ), while β-Arrestin-1 downregulated H4 acetylation in the promoter regions of *HDAC7A*, *APO2*, and *TRAIL* in autoreactive T cell line (Table 1) (Hu et al., 2011). Thus, under the regulation of histone modifications, overexpression of β-Arrestin-1 might contribute to the pathogenesis of PBC *via* increased T cell proliferation, augmented interferon production, downregulated activities of NF-κB and activating protein-1 (AP-1) (Hu et al., 2011). Besides, a recent study showed that H3K27ac and transcription factor ETS1 were increased at the promoter of the anti-apoptotic gene B-cell lymphoma-extra large (*BCL-xL*) during cholangiocyte senescence, which is important in PSC pathogenesis (Table 1; Figure 2) (O’hara et al., 2019). Cyclin-dependent kinase inhibitor 2A (CDKN2A) is a known tumor suppressor gene that inhibits cell growth and inhibits tumors (Padhi et al., 2017). Increased ETS1 and H3K4me3 at the *CDKN2A* promoter was also found in cholangiocyte senescence (Table 1; Figure 2) (O’hara et al., 2017). Therefore, increased H3K27ac and H3K4me3 upregulate the expression of genes which are crucial in cholangiocyte senescence, contributing to the development of PBC (Figure 3E). Together, there is little research on the mechanism of histone modification in autoimmune liver disease, and more research is urgently needed.

### 3.5 Drug-Induced Liver Injury

Drug-induced liver injury (DILI) is one of the most common causes of acute liver failure and is typically classified as direct, indirect, and idiosyncratic injury (Hoofnagle and Björnsson, 2019). Direct injury is generally caused by a high or cumulative dose of intrinsically toxic agents and is predictable (Hoofnagle and Björnsson, 2019). Nevertheless, indirect injury is caused by the action of the agents in the liver rather than by its toxicity or properties (Hoofnagle and Björnsson, 2019). The idiosyncratic injury occurs in rare cases following exposure to a therapeutic dose of agents with no intrinsic toxicity for a standard duration, and is often unpredictable (Krueger et al., 2014). The pathogenesis of DILI is not well understood. Covalent binding between macromolecules and drug metabolites, inhibition of the bile acid transport system, intracellular ion imbalance, mitochondrial respiratory dysfunction, oxidative stress and stimulation of the intrinsic immune system are reported to be involved in the pathogenesis of DILI (Lee et al., 2016; Hoppmann et al., 2020). Some other theories have suggested that the pathogenesis of DILI may also involve genetic polymorphism and epigenetic modifications (Krueger et al., 2014). A study of TAA-treated mice showed that the H3K9ac level was increased during the initial injury phase and decreased subsequently, which might count for the activation of stress-related genes (Table 1; Figure 2) (Sinha et al., 2016). Besides, H3K9me3 signal was found to increase after injury and maintained up for a short time, followed by a gradual decline, suggesting H3K9me3 might be responsible for the repression of some tress and metabolic-associated genes (Table 1; Figure 2) (Sinha et al., 2016). Moreover, H3S10ph was found absent in the injury phase but increased sharply in the regeneration phase, indicating that H3S10ph may play a critical role in liver regeneration after injury (Table 1; Figure 2) (Sinha et al., 2016). However, this study did not identify genes of which the expression was affected by histone modifications. In conclusion, little is known about the role of histone modification in DILI, and future studies need to address this issue.

### 3.6 Liver Fibrosis/Cirrhosis

Liver fibrosis and cirrhosis are the end-stage of all the above-mentioned CLDs (Roehlen et al., 2020). Excessive extracellular matrix (ECM) proteins deposition due to increased synthesis and decreased degradation eventually leads to scar tissue formation, liver fibrosis, and liver cirrhosis (Mahdinloo et al., 2020). Activated hepatic stellate cells (HSCs) are identified as the major ECM-producing cells in injured liver (Bataller and Brenner, 2005). Besides, hepatocytes, LSECs, Kupffer cells, cholangiocytes and recruited cell types (*e.g*., bone-marrow recruited macrophages) also contribute to liver fibrosis (Kisseleva and Brenner, 2021; Qing et al., 2021). The mechanisms of liver fibrosis and cirrhosis are complex, involving different cells, signaling pathways, and liver microenvironment. Nevertheless, the development of epigenetic research has uncovered the role of histone modifications in liver fibrosis and cirrhosis.

#### 3.6.1 Histone Acetylation in Liver Fibrosis/Cirrhosis

Previous studies have demonstrated that H3K9 acetylation on the promoter of *TNFα* and epidermal growth factor receptor (EGFR) increases MYC (the super-transcription factor) and cyclin D1 (the cell cycle regulator) expression (Yang et al., 2014; Sarr et al., 2016; Zheng et al., 2020). These changes subsequently activate HSCs, promoting the development of liver fibrosis (Table 1) (Yang et al., 2014; Sarr et al., 2016; Zheng et al., 2020). These studies suggest that H3K9 acetylation contributes to fibrogenesis, while the signaling pathway between the H3K9ac-upregulated gene expression and the resulting HSC activation is not well characterized. Besides, increased levels of H3K27ac in active HSCs were found at regulatory elements and enhancers of the AP-1 motif (TGACTCA), which is a known driver of HSC activation (Liu et al., 2020). Moreover, increased H3K27ac level at regulatory elements of Collagen 1α1 (Col1α1) and Collagen 1α2 (Col1α2) and enhancers of the transcriptional enhanced associate domain (*TEAD*) and NF-κB motifs were also observed in active HSCs (Table 1) (Liu et al., 2020). TEAD, a transcription factor, integrates and coordinates multiple signal transduction pathways, including EGFR and transforming growth factor beta (TGF-β) signaling pathways (Huh et al., 2019). Moreover, TGF-β and synectin (known as GIPC) were found to promote HSC activation and migration (Yaqoob et al., 2020). TGF-β and GIPC treatment increased H3K27 acetylation and decreased H3K27 trimethylation at the promoter of the insulin-like growth factor binding protein-3 (IGFBP-3), which is involved in cellular differentiation and migration, in HSCs (Yaqoob et al., 2020; Chao et al., 2021). These results suggest that H3K27 acetylation plays an important role in collagen deposition, inflammatory response, and HSC activation and migration during liver fibrosis *via* upregulating the abovementioned genes and possibly involving EGFR and TGFβ signaling pathways. BRD4 is a reader of H3K27ac, and BRD4 is highly enriched at enhancers of profibrotic genes of activated HSCs (Table 1; Figure 2) (Ding et al., 2015). Thus, BRD4 inhibitor JQ1 could attenuate liver fibrosis in a carbon tetrachloride (CCl_4_)-induced fibrotic murine model (Ding et al., 2015). On the contrary, the acetylation of H4 at the antifibrogenic factor PPARγ gene was depleted during liver cirrhosis (Rodríguez-Aguilera et al., 2018). Treatment with a histone deacetylase inhibitor, valproic acid (VPA), increased histone H4 acetylation and inhibited proliferation of primary murine HSCs (Mannaerts et al., 2010). Besides, inhibition of HDAC2 was also found to reduce liver fibrosis in a minipig NASH model (Table 1; Figure 2) (Zhang et al., 2021). Collectively, it seems that the pro-fibrosis genes are usually accompanied by the activation of histone acetylation, while the anti-fibrosis genes are often shown as the absence of acetylation in the process of liver fibrosis (Alonso-Merino et al., 2016). Interestingly, a previous study found that liver injury in male ancestors reduces liver fibrogenesis in F2 male offspring (Zeybel et al., 2012). This intergenerational adaptation is attributed to heritable reprogramming of histone H3 acetylation in *P*PARγ and *TG*F-β1 (Zeybel et al., 2012). However, prospective epigenetic studies in families with liver disease are required to test this hypothesis. Apart from the effect of histone acetylation on gene expression, a recent study pointed out that histone acetylation could also regulate the expression of miRNAs in HSCs (Lu et al., 2019).

LSECs are another kind of specialized endothelial cell in the liver. p300/NFκB/BRD4 protein complex promoted *CCL2* transcription *via* increasing H3K27ac at enhancer and promoter regions in LSECs (Table 1; Figure 2) (Gao et al., 2021). LSEC-specific p300 deletion or BRD4 inhibitor could significantly attenuate macrophage infiltration and liver fibrosis (Gao et al., 2021). Altogether, it seems that activation of histone acetylation in liver cells increases expression of pro-inflammatory and pro-fibrotic genes, leading to the progression of liver fibrosis (Figure 3F).

#### 3.6.2 Histone Methylation in Liver Fibrosis/Cirrhosis

During the transdifferentiating process of HSCs, fibrogenic genes such as alpha-smooth muscle actin (αSM*A*), tissue inhibitor of metalloproteinase-1 (*TIMP1*), *collagen I*, and TGF-β1 were upregulated via ASH1 mediated hypermethylation of H3K4 (Table 1; Figure 2) (Perugorria et al., 2012). This suggests that H3K4 methylation is important for the transformation of HSC into myofibroblasts. In general, multiple sites of histone methylation modify together to regulate gene expression. For example, the *TGF-*β overexpression in the bile duct ligation model of rats was accompanied by upregulated levels of active transcription marks (H3K4me1, H3K4me2, and H3K4me3) and reduced levels of suppressive marks (H3K9me2 and H3K9me3) in the promoter of *TGF-*β (Sheen-Chen et al., 2014). Apart from the abovementioned genes, multiple genes are regulated by histone methylation during liver fibrosis. H3K27me3, a transcripton silencing mark, was enriched in the promoter of the antifibrogenic factor *PPAR*γ in rats with liver fibrosis, and it was associated with the progression of liver disease (Table 1) (Zeybel et al., 2012). Besides, the Hypoxia-inducible factor-1 (HIF-1) transcriptional complex was associated with an increased level of transcription activating mark H3K4me3 (Hong et al., 2018). This increased H3K4me3 is related to HIF-1 nuclear transport, autophagosome formation, and activation of HSC *in vitro* (Table 1) (Hong et al., 2018). Moreover, enhanced autophagy in homocysteine (Hcy)-treated hepatocytes was mediated by reduced expression of cystic fibrosis transmembrane conductance regulator (CFTR). Likewise, *CFTR* was regulated by the increased H3K27me3 level at the *CFTR* promoter, which was catalyzed by histone methyltransferase zeste homolog 2 (EZH2) (Table 1; Figure 2) (Yang et al., 2018). Altogether, histone methylation of histones permits the expression of fibrogenic genes and inhibits the expression of protective genes, promoting the progression of liver fibrosis (Figure 3F).

#### 3.6.3 Other Histone Modifications in Liver Fibrosis/Cirrhosis

Currently, there are few studies on the role of histone phosphorylation in liver fibrosis, but the changes and functions of histone phosphorylation in the above-mentioned CLDs might also work for liver fibrosis. Nevertheless, histone ubiquitination may be involved in the process of liver fibrosis. It is known that monoubiquitination of H2A and H2B can influence the methylation state of histone H3, regulating the activation or repression of gene expression, which suggests that histone ubiquitination can indirectly regulate disease progression via crosstalk with H3 methylation (Li et al., 2017). Besides, ring finger protein 20 (RNF20), also known as E3 ubiquitin-protein ligase BRE1A, has been demonstrated to inactivate IL-6, TNF-α, VEGFA, α-SMA as well as collagen I and alleviate liver fibrosis via ubiquitination of H2BK120 (H2BK120ub) *in vitro* and *in vivo* (Table 1; Figure 2) (Chen et al., 2021). These results imply that ubiquitination of H2B may play a protective role in liver fibrosis, while the underline mechanisms need further investigation. BRCA1-Associated Protein 1 (BAP1) is a major tumor suppressor and belongs to the deubiquitinase superfamily (Masclef et al., 2021). BAP1 mutations were discovered in intrahepatic cholangiocarcinoma and hepatocellular carcinoma (Masclef et al., 2021). Moreover, a previous study assumed that BAP1 modulated gene expression by inhibiting H2A ubiquitination, and BAP1 inactivation led to apoptosis of fibroblasts and liver tissue in mice (Table 1; Figure 2) (He et al., 2019). Therefore, there might be a possibility that H2A ubiquitination plays a role in tumorigenesis. However, this hypothesis requires multiple studies to explore the ordered molecular events leading to malignant transformation after BAP1 inactivation. Taken together, acetylation, methylation, and ubiquitination of histones contribute to liver fibrosis and cirrhosis by inhibiting the transcription of protective genes and activating the pro-inflammatory and fibrogenic genes (Figure 3F). However, further studies are still needed to reveal the specific histone modifications at relevant genes and their corresponding enzymes to develop potential drugs.

## 4 Therapeutic Opportunities

### 4.1 Lifestyle Changes

It was reported that physical exercises and lifestyle changes could gradually modulate and reverse the epigenetic changes (Sodum et al., 2021). Besides, the Mediterranean diet, which is characterized by reduced carbohydrate intake and increased omega-3 and monounsaturated fatty acid intake, is the most recommended dietary pattern for MAFLD (Romero-Gómez et al., 2017). Therefore, at the early stages of liver diseases, lifestyle changes and physical exercises might be the future advancements in treating metabolic disorders.

### 4.2 Potential Targets

Histone modifications depend primarily on the substrates, writers, erasers, and readers. Therefore, the acetyl-donor, methyl-donor, HDACs, HACs, HMTs, HDMTs, and the “readers” all can act as the potential target for the treatment of CLDs (Tables 1, 2 and Figures 2, 3). The existing studies on histone modification are mainly aimed at malignant diseases such as liver cancer, and there are few studies on the therapeutic effects of benign liver diseases. Acetyl-CoA was the acetyl-donor in the histone acetylation reaction. Pyruvate dehydrogenase complex (PDHC) and lactate dehydrogenase (LDH) would translocate to the nucleus and increase the nuclear concentrations of acetyl-CoA (Ferriero et al., 2018). Acetyl-CoA subsequently, results in hyper-acetylation of histone H3 and promotes the expression of damage response genes (Ferriero et al., 2018). Besides, treatment with the LDH inhibitor alleviated liver damage and improved survival in hepatotoxins induced acute liver failure (Ferriero et al., 2018). Therefore, the LDH inhibitor, which reduced the acetyl-donor, maybe a new target for the treatment of CLDs. SIRT1, which belongs to HDAC III, enhances HBV replication by targeting the transcription factor AP-1 (Li et al., 2016). The SIRT1 inhibitor nicotinamide inhibits HBV replication both *in vitro* and *in vivo* (Li et al., 2016). Moreover, HDAC III inhibitor treatment was reported to block HCV replication in a mouse model (Zhou et al., 2018). These studies imply that HDAC III inhibitor may be a new treatment strategy for viral hepatitis. Interestingly, SIRT1 is downregulated during organ injury and aging-associated fibrosis (Han et al., 2021), which means SIRT1 can also be a new therapeutic target in liver fibrosis. Chromatin reader BRD4 is the reader of H3K27ac, and the BRD4 inhibitor iBET-151 could ameliorate hepatic inflammation and liver injuries in CCl_4_-induced acute liver injury and alcoholic hepatitis (Gao et al., 2021; Liu et al., 2021). Therefore, the histone acetylation readers could also be a potential target for the treatment of CLD. In short, the regulation of histone acetylation can be a promising direction for the treatment of CLDs (Tables 1, 2 and Figures 2, 3).

**TABLE 2:** Summary of potential therapeutic targets and the corresponding clinical approved/non-approved drugs.

Category	Therapeutic targets	PDB-ID	Function	Clinical/non clinical approved drugs	References
Writers	P300	5LKU	Acetylates histone lysines	C646, B026, A-485, B029-2, etc.	Lasko et al. (2017), Peng et al. (2019), Yang et al. (2020), Cai et al. (2021)
	ASH1	3MQM	Methylates histone H3K36 and H3K4	—	Perugorria et al. (2012)
	EZH2	4MI5	Methylates histone H3K27	Tazemetostat; Valemetostat, GSK126, CPI-0209, CPI-1205	Huang et al. (2019), Bates (2020)
	SETDB1	3DLM	Methylates histone H3K9	Mithramycin, DZNep	Rivière et al. (2015), Karanth et al. (2017)
	MLL4/KMT2D, SET1A	4Z4P, 4RIQ	Methylates histone H3K4	Methyltransferase inhibitor 5′-deoxy-5'-(methylthio)adenosine (MTA)	El Mansouri et al. (2011), Kim et al. (2016)
	SET7/9 (KMT7)	1N6A	Methylates histone lysines	MTA, Cyproheptadine, Sinefungin, etc.	Gu et al. (2018), Tamura et al. (2018); Sharma et al. (2020)
	PRMT1, PRMT5	1OR8, 6UXX	Methylates histone arginine	EPZ015666, GSK33265 95, GSK591, LLY-283, etc.	Zhang et al. (2017), Guccione and Richard (2019)
	RNF20/BRE1A	5TRB	Ubiquitination of H2B	—	Chen et al. (2021)
Erasers	HDAC	—	Deacetylase removes acetyl groups from histone lysines	Vorinostat, belinostat, panobinostat, romidepsin; Fimepinostat, KA2507, OKI-179, etc.	Rodríguez-Aguilera et al. (2018), Bates (2020)
	LSD 1	4XBF	Demethylates histone lysines	Iadademstat, CC-90011, INCB059872	Alarcon et al. (2016), Bates (2020)
	JMJD2B/KDM4D	4UC4	Demethylates histone lysines	JIB04, Curcuminoids	Kim et al. (2014), Kurozumi et al. (2019), Kim et al. (2020)
	Phf2	3PTR	Demethylates histone lysines	—	Bricambert et al. (2018)
	BAP1	—	Deubiquitination of H2B	—	He et al. (2019)
Readers	BRD4	5EI4/5EGU	Bromodomain proteins read acetyl groups on histone lysines	Molibresib, Birabresib, ZEN003694, PLX51107	Bates (2020), Gao et al. (2021)

As we mentioned before, SAM, the methy-donor, can attenuate the alcohol-induced liver injury (Bardag-Gorce et al., 2010), suggesting that the SAM supplement can be a novel treatment for ALD. EZH2 specifically catalyzes the methylation of H3K27, which results in chromatin compaction and gene silencing (Lim and Kim, 2020). It has been proved that EZH2 plays a critical role in liver inflammation and liver fibrosis and eventually drives MAFLD progression (Lim and Kim, 2020). EZH2 inhibitor (DZNep) was found to inhibit multiple histone methylation and was sufficient to suppress liver fibrosis by modulating histone methylation of HSC in a mouse model (Zeybel et al., 2017). Moreover, the demethylase JMJD3 has a similar effect as the DZNep (Jiang et al., 2021). Therefore, the HMTs and HDMTs, which regulate the methylation of histones, can be new targets for the treatment of liver fibrosis (Tables 1, 2; Figures 2, 3). However, few studies focus on histone phosphorylation as a treatment in CLD, and more preclinical studies are needed in the future.

Inspiringly, several drugs such as belinostat, abexinostat, romidepsin, and vorinostat were ongoing for the treatment of T-cell lymphoma, and panobinostat for multiple myeloma (Yeo et al., 2012; Eckschlager et al., 2017). However, because of the non-significant efficacy and the certain toxic and side effects, these drugs are generally used in combination with other chemotherapy drugs (Eckschlager et al., 2017; Takebe et al., 2019; Shetty et al., 2021). In the context of CLDs, the lack of deep mechanism studies and well-designed clinical trials impedes the application of histone modification inhibitors in CLD.

## 5 Current Challenges and Prospects

We summarized the current findings of histone modifications in CLDs in Tables 1, 2 and Figures 2, 3. Apart from the abovementioned observations, there are still some issues that remain unclear and need further studies. 1) The previous studies found that histone modifications play a pivotal role in the occurrence and progression of CLDs, and the modifications can be inherited or produce intergenerational adaptation. However, the transgenerational and epigenetic mechanisms in CLD need to be verified by more prospective population studies. 2) In a specific disease, different sites of histone modifications play different roles, and the cumulative effect of multiple histone modifications ultimately evokes the transcriptional activity of a gene. Besides, the histone modifications collaborate with transcription factors and “readers” to determine the transcription of target genes. Therefore, it is challenging to clarify the effect of a particular modification on the development of CLDs. 3) Many proteins regulate and interpret the PTMs, including readers, writers, and erasers. The specific inhibitors that targeting these proteins might serve as therapeutic agents for ALD. However, these PTMs regulatory proteins could modify histone modifications in dozens of genes without specificity. The off-target effect should be avoided while developing the inhibitor targeting these proteins. 4) The hepatocytes, HSCs, LSECs, Kupffer cells, and cholangiocytes in the liver cooperatively regulate hepatic function and contribute to CLDs. The complex of histone modifications in liver cells and their cross-talk also deserves to be manifested in the future. The single-cell sequencing, chromatin immunoprecipitation sequencing, and other advanced molecular technologies might provide tools for us to explore the mechanism underlying histone modification in CLDs.

In conclusion, histone modifications are one of the most revolutionary targets in the treatment of various human diseases. Histone modifications, different from genetic mutations, are reversible and can be pharmacologically modulated. Some epigenetic drugs have been approved for clinical use, and several compounds are currently in stages of preclinical drug development. Thus, selectively tissue-targeted drugs with low toxicity and high efficacy are required to completely reverse CLDs and prevent the development of liver cirrhosis and malignancy.
